# Gastrointestinal stromal tumor: Role of surgery and immunotherapy

**DOI:** 10.4103/0971-9261.72442

**Published:** 2010

**Authors:** Sushmita N. Bhatnagar

**Affiliations:** Department of Pediatric Surgery, B.J. Wadia Hospital for Children, Parel, Mumbai, Maharashtra, India

**Keywords:** Gastrointestinal stromal tumor, immunotherapy, primary surgery

## Abstract

Report of a gastrointestinal stromal tumor in an 11-year-old girl who presented with a large lump in the upper abdomen. After complete surgical excision and histopathology, postoperative immunotherapy with imatinib led to an excellent outcome and a tumor-free survival of 3 years.

## INTRODUCTION

Gastrointestinal stromal tumor (GIST) is an entity in the group of mesenchymal tumors.[1] For many years, surgery was the only modality available as chemotherapy and radiotherapy were largely ineffective. With the introduction of “molecular targeted therapy” imatinib mesylate in the treatment of GIST in the year 2000, the outcome of treatment of this tumor improved to a great extent. The initial use of this drug was solely for the advanced, metastatic disease, but over the last 10 years, its use has evolved to adjuvant therapy and, recently, to neoadjuvant therapy. Reported here is an 11-year-old girl with GIST who was successfully treated with surgery and adjuvant imatinib therapy.

## CASE REPORT

**Figure 1:**
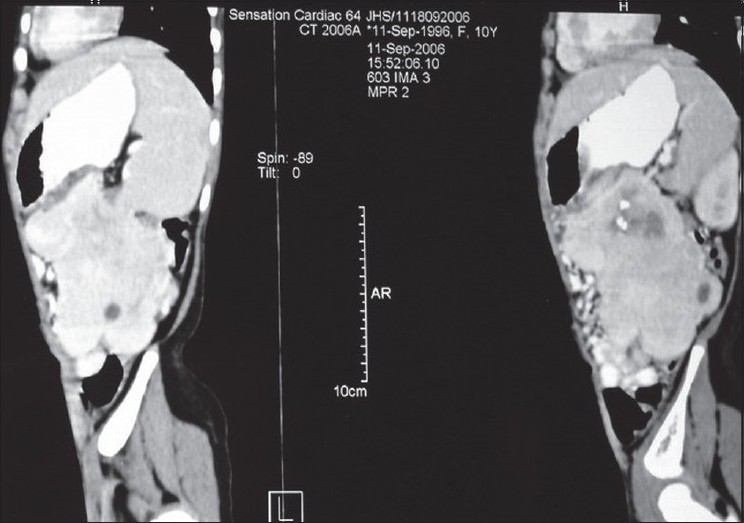
Computed tomography scan of the abdomen showing the large tumor inferior to the stomach and displacing the stomach and colon

**Figure 2:**
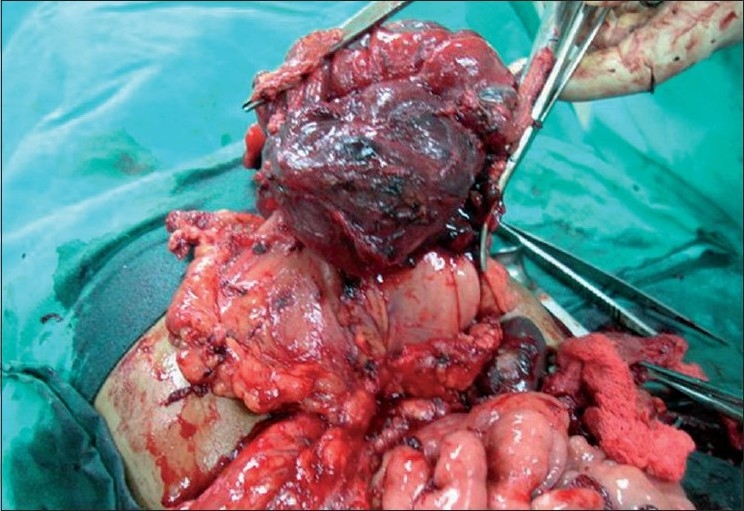
Intraoperative photograph showing tumor arising from the greater curvature and involving the transverse colon, which has been resected along with the mass

An 11-year-old girl presented with complaints of low-grade intermittent fever for 6 months and a gradually increasing lump in the left upper abdomen of 15 days duration. On examination, a 10 cm × 5 cm nontender, firm, freely mobile, mass with ill-defined margins was palpable in the left hypochondrium. The investigations revealed anemia and raised Lactate Dehydrogenase. Alfa-fetoprotein and a chest radiograph were normal. A computed tomogram (CT) scan showed a large lobulated heterogeneously enhancing mass of size 11 cm × 6 cm × 7 cm, with central area of necrosis and calcification in left hypochondrium and lumbar region [Figure 1]. The mass was situated anterior to the descending colon and indenting the posterior wall of the stomach, displacing the small bowel loop superiorly and medially with no involvement of the major blood vessels, most probable diagnosis being a teratoma. Intraoperatively, a 11 cm × 6 cm × 5 cm mass was found arising from the posterior wall of the stomach, infiltrating into the transverse mesocolon and pancreatic bed and adherent to the spleen. The mesenteric lymph nodes were enlarged, four of which were excised and hemorrhagic ascites (50 ml) were sent for cytology. Gross total resection of the mass was performed. The resected specimen included part of the posterior wall of the stomach near the greater curvature and about 5” of the transverse colon [Figure 2]. Colo-colic anastomosis and repair of the stomach in two layers with feeding jejunostomy was carried out with the intention of starting early feeds. Jejunostomy feeding was started on day 3 and oral feeding commenced on day 10 postoperatively. The histopathology revealed gastrointestinal tumor with predominant epitheloid cells focally positive for CD34 and C-kit gene, which is diagnostic. KIT mutational studies are not available in India and hence could not be performed. The mitotic rate of the tumor was <5/high-power field. Surgical margins, lymph nodes, omentum and ascitic fluid were negative for tumor cells. As the tumor was more than 10 cm and positive for c-kit gene, the child was given adjuvant therapy of imatinib 260 mg/m^2^ a day. The dose was then reduced to 200 mg/m^2^ as the child had jaundice, with a serum bilirubin of 2.2 mg/dl after the first month. This was continued for 6 months. The child was followed with ultrasonography scans at regular intervals and, at the 3-year follow-up, is well and tumor-free.

## DISCUSSION

GISTs are a type of mesenchymal tumors [Table 1].[2] With the recent advances and reports of a large number of pediatric patients with GIST [Table 2],[3–6] it is now clear that the characteristics of this tumor are not comparable to the adult counterparts as the clinical behavior and the potential for malignancy of this tumor is variable and thus the prognostication becomes difficult. Preoperative biopsy is generally not recommended as per the consensus guidelines due to the risk of hemorrhage and tumor spillage.[7] In the past, the primary treatment modality was surgery, and adjuvant therapies like chemotherapy and radiotherapy were not recommended as in other tumors due to their ineffectivity.

**Table 1:** Tumor types with differential diagnosis of GIST

Smooth muscle	Leiomyoma leiomyosarcoma (LMS)
Neural tissue	Schwannoma
	Malignant Peripheral Nerve Sheath Tumor (MPNST)
	Neurofibroma
	Neuroendocrine Tumor Carcinoid
	Carcinosarcoma
Connective tissue cells	Fibromatosis Or Desmoid Tumor
	Solitary Fibrous Tumor
	Inflammatory Fibroid Polyp
Others	Angiosarcoma
	Clear Cell Sarcoma
	Liposarcoma
	Synovial Sarcoma
	Malignant Mesothelioma
	Dedifferentiated Carcinoma
	Sarcomatoid Carcinoma
	Metastatic Melanoma

**Table 2:** Differentiating features between Pediatric and Adult GIST(14)

Feature	Pediatric GIST	Adult GIST
Gender distribution	>Common in girls than in boys	Almost equal incidence with male preponderance
Spread	Spreads to lymph nodes in about 30%	Lymph node involvement rare (1-2%)
Recurrence pattern	>likely to recur in the original location	<likely
Metastases	> likely to metastasize (23%)	< likely to metastasize (2.5%)
	Mets to liver more common	
	If co-existing Carney Triad, metastases more likely	
Course	Course of disease less aggressive	Even with less advanced disease, GIST can be very aggressive
Genetic pattern	Differing gene mutations and differing gene expressions	Exon 11 mutations >common
	If mutations exist, Exon 9 mutations are >common	

Even with advanced GIST where complete resection was not possible and/or extensive resection of the structures/organs involved, surgical excision used to be the main treatment modality.[8] With the introduction of “imatinib mesylate” for the treatment of GIST in 2000, and the report of successful management of advanced disease by this drug in 2001,[9] the role of this oral drug therapy has been gradually evolving. In 2002, the Food and Drug Administration (USA) approved this drug for use in advanced metastatic and unresectable GIST,[10] and its efficacy was established in a study of 147 adult patients. Subsequently, this drug found increasing application for both adult and pediatric GISTs as an adjuvant therapy[11,12] thus improving the overall tumor-free survival and much reduced recurrence of this subset of patients.

The enthusiasm of the use of this drug led to its utilization as neo-adjuvant therapy in adults. An Radiation Therapy Oncology Group trial in adults with large or marginally resectable tumors[13,14] implemented a selection criteria of patients with primary tumor more than or equal to 5 cm, recurrent tumor of 2 cm or more and a potentially resectable mass. As neoadjuvant therapy, its purpose was to reduce the extent and morbidity of the surgical intervention. As yet, imatinib mesylate has not found usage as neo-adjuvant therapy for all children with GIST as upfront therapy (similar to the International Society of Paediatric Oncology protocol of upfront chemotherapy in Wilms’ tumor). The child reported here had a large-sized tumor, more than 10 cm, and had involvement/infiltration of the transverse colon, which had to be resected. Large size of the tumor and an unpredictable behavior of such tumors prompted the pediatric oncologists to give a course of immunomodulator therapy with imatinib, which was tolerated well and provided a tumor-free follow-up period of 3 years. In lieu of complete resection, the dose of imatinib selected was 260 mg/m^2^, which was reduced to 200 mg/m^2^ in view of hepatic impairment. There is no consensus on the duration of treatment with imatinib and further research and study of a large number of cases will provide future guidelines.

Pediatric GISTs are still classified, risk stratified and treated according to the adult GIST protocols. As more and more patients will be managed and reported, the understanding of pediatric GISTs (which has a different behavior from its adult counterpart) will increase and thus expand the scope of multimodality treatment. Surgical excision is and shall continue to play a cardinal role in the management protocol of this uncommon pediatric tumor.
